# Supplementary material for: Driving forces behind phase separation of the carboxy-terminal domain of RNA polymerase II
Source: Nat Commun. 2023 Sep 25;14:5979. doi: 10.1038/s41467-023-41633-8 (PMC10519987; doi:10.1038/s41467-023-41633-8)
Supplement: Supplementary file 1 — Supplementary Information [file 41467_2023_41633_MOESM1_ESM.pdf]

Supplementary information

Table of contents

Supplementary figures

Supplementary Figures

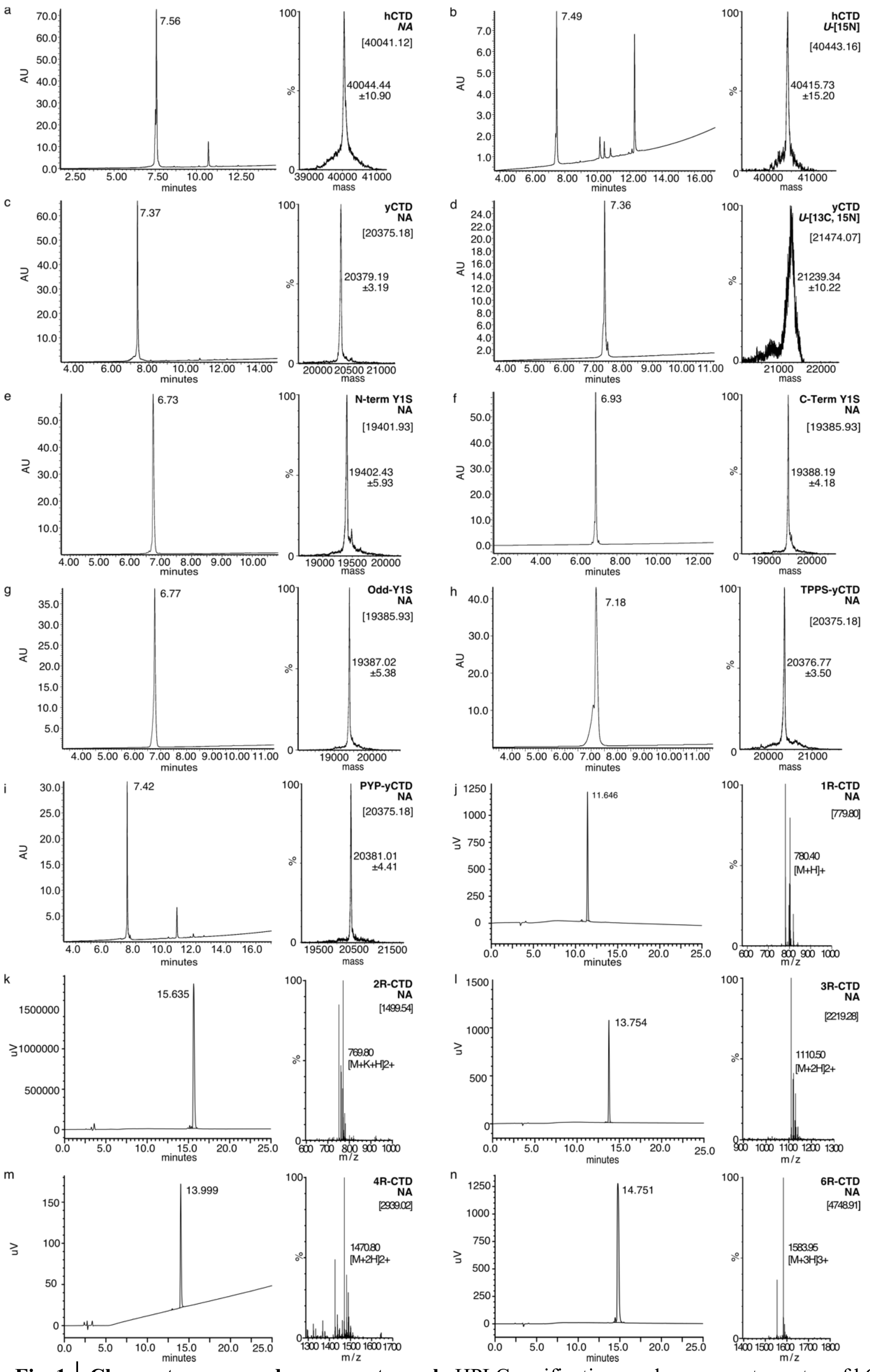

Supplementary Fig. 1 │ Chromatograms and mass spectra. a-h, HPLC purifications and mass spectrometry of hCTD (panels a-b), yCTD (c-d), variants (e-i) and model peptides (j-n). Retention times for the prominent peaks are indicated in the chromatograms (left part of the panels). Mass spectra (right side of each panel) indicate the labeling scheme (^{15}N, ^{13}C or NA: natural abundance) and the theoretical mass in squared parenthesis. Masses from the prominent peak are indicated next to the deconvoluted peak (hCTD, yCTD & variants) or the charged ion (peptides).

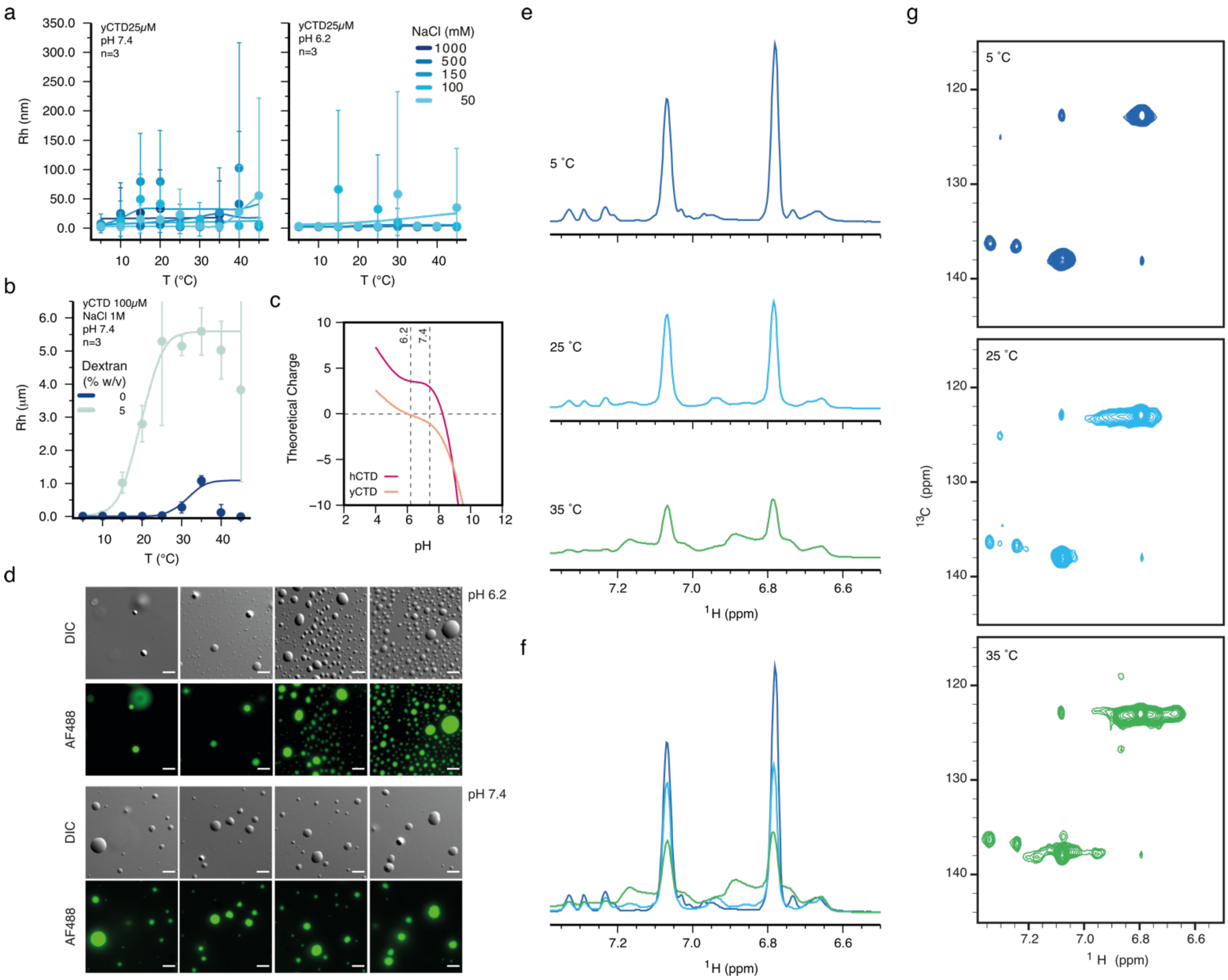

Supplementary Fig. 2 │ Phase separation of yCTD. a, Influence of temperature, pH, and ionic strength to induce higher order aggregate formation of yCTD (25 µM yCTD in 25 mM HEPES, 1.0 mM TCEP) monitored by dynamic light scattering. Dots represent mean values for independent measurements (n=3). Solid lines represent sigmoidal regression. Error bars represent two times std. **b,** Phase separation of yCTD at higher protein concentration (100 µM) and increased ionic strength (1 M NaCl) monitored by dynamic light scattering in the absence of a molecular crowding agent (blue) or with 5 % w/v dextran (cyan). Error bars represent two times std for mean values of independent measurements (n=3). **c,** Theoretical net charge of hCTD (red) and yCTD (orange) as a function of pH. **d,** DIC and fluorescence microscopy of yCTD (100 µM yCTD in 25 mM HEPES, 1 M NaCl, 1.0 mM TCEP) phase separation at pH 6.2 (top) and pH 7.4 (bottom). Droplet formation was promoted with dextran (5% w/v; see (b)). **e-f,** Tyrosine ring resonances in one-dimensional projections from the 2D ^{1}H-^{1}H NOESY experiments shown in Fig. 1d. The superposition of the projections is shown in (f). **g,** Aromatic regions from two-dimensional ^{1}H-^{13}C-HSQC spectra of yCTD in the same conditions in which the ^{1}H-^{1}H NOESY experiments of Fig. 1d were recorded. At 25 ˚C and 35 ˚C, the sample visually formed a condensate in the NMR tube. Micrographs are representative of 3 independent biological replicates. Scale bar, 5 µm**.**

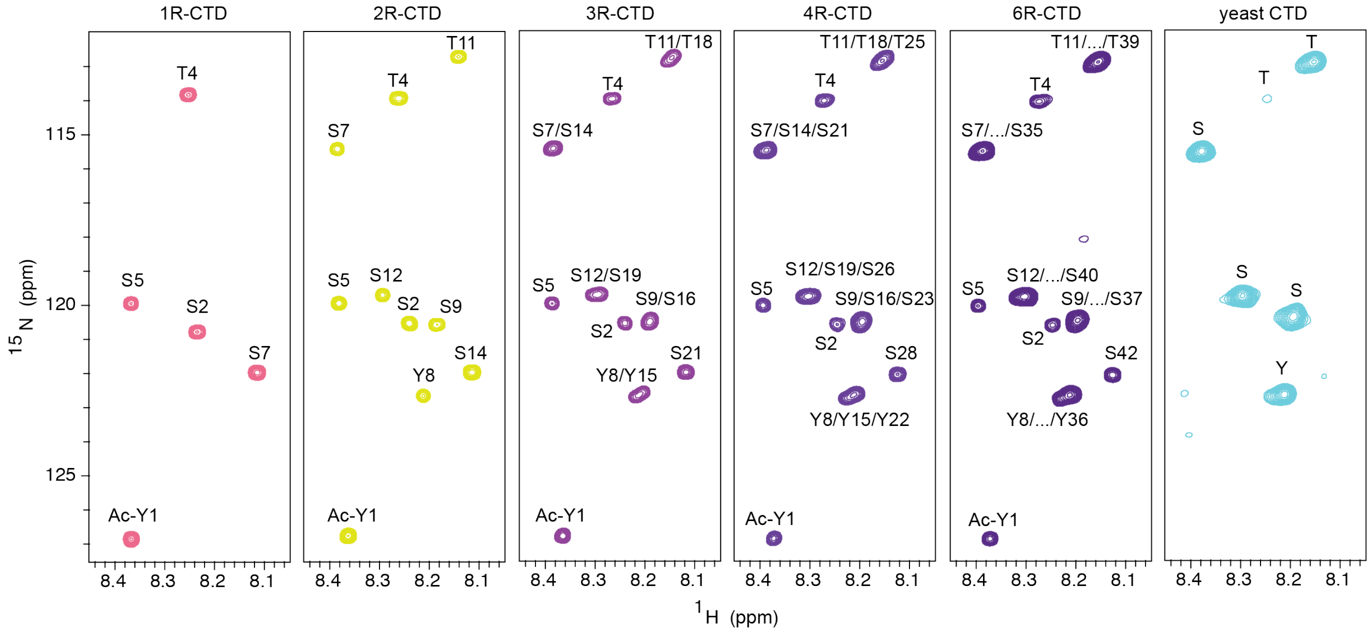

Supplementary Fig. 3 | Canonical YSPTSPS repeats have a similar chemical environment. ^{1}H-^{15}N HSQC spectra for yCTD and CTD peptides composed of one to six canonical YSPTSPS repeats. yCTD was uniformly ^{13}C/^{15}N-labelled; CTD peptide spectra were recorded at natural abundance. Sequence-specific resonances assignments obtained for 1R-CTD, 2R-CTD and 3R-CTD are indicated. For 4R-CTD and 6R-CTD the chemical shift degeneracy is reflected by additional assignment labels (e.g. S21, T25, …).

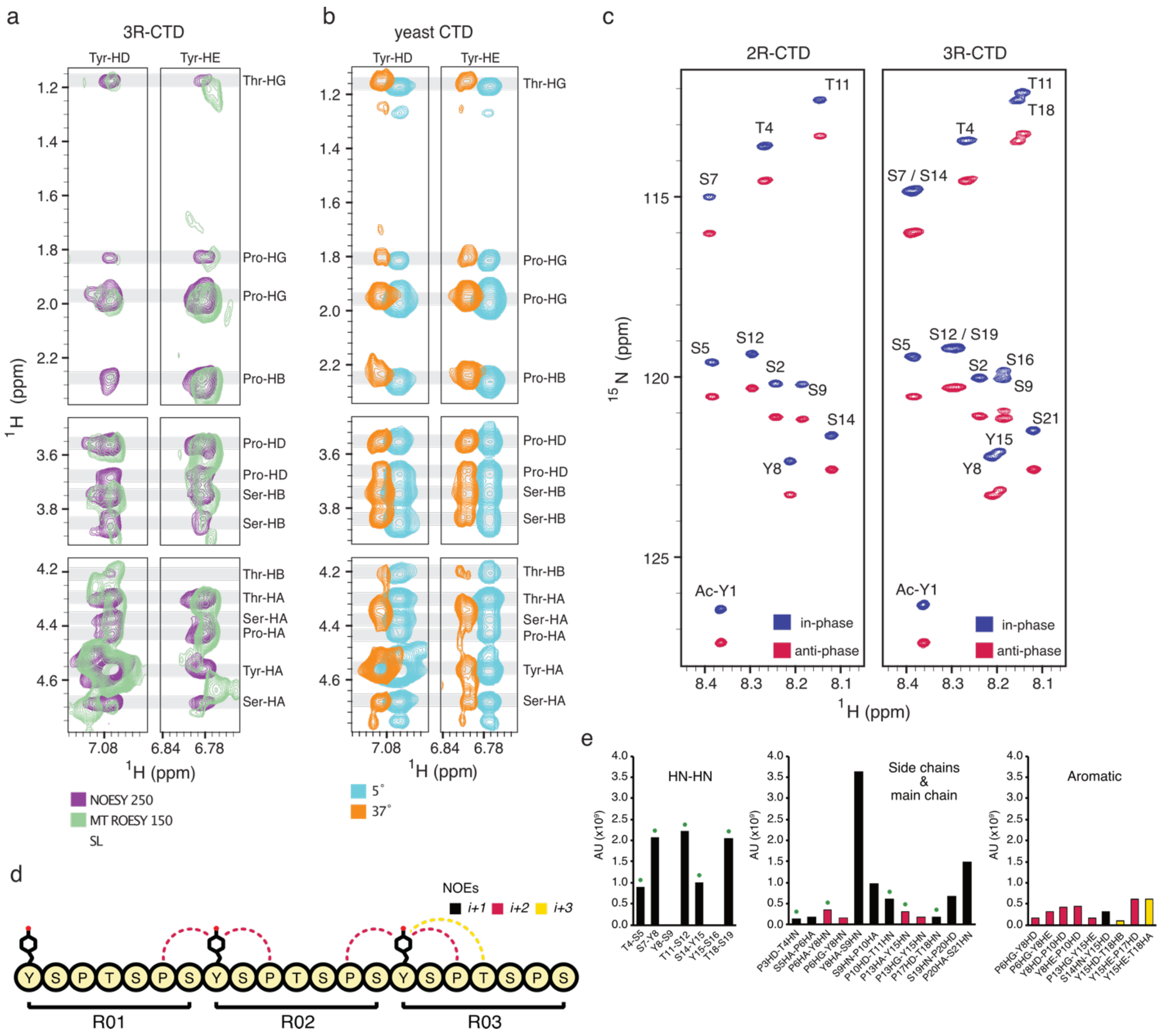

Supplementary Fig. 4 | Tyrosine-Proline contacts shape CTD structure. **a,** Superposition of the aromatic side-chain region from the two-dimensional ^{1}H-^{1}H NOESY (mixing time 250 ms; purple) and two-dimensional ^{1}H-^{1}H ROESY (spin lock 150 ms; green) experiments for 3R-CTD at 5 °C. **b,** The overlay of the aromatic side-chain region from the two-dimensional ^{1}H-^{1}H NOESY experiments (mixing time 250 ms) of yCTD at 5 °C (cyan) and 37 °C (orange). **c,** Superimposed in-phase and anti-phase components from the gradient-enhanced IPAP-HSQC experiments recorded for 2R-CTD and 3R-CTD peptides. The spectra were recorded with high resolution in the ^{15}N dimension to decrease signal overlap. **d,** Schematic representation of the detected medium-range NOE-contacts in 3R-CTD. **e,** Sequential and medium range NOEs in 3R-CTD. NOEs in the left part of the panel highlight HN-HN interactions, while the central and the right graphs show atom pairs between combinations of side chain-main chain and aromatic rings. Green circles above the bars in arbitrary units (AU) indicate a propensity for β-turns, according to the work published by M.M. Harding (DOI: https://doi.org/10.1021/jm00103a002).

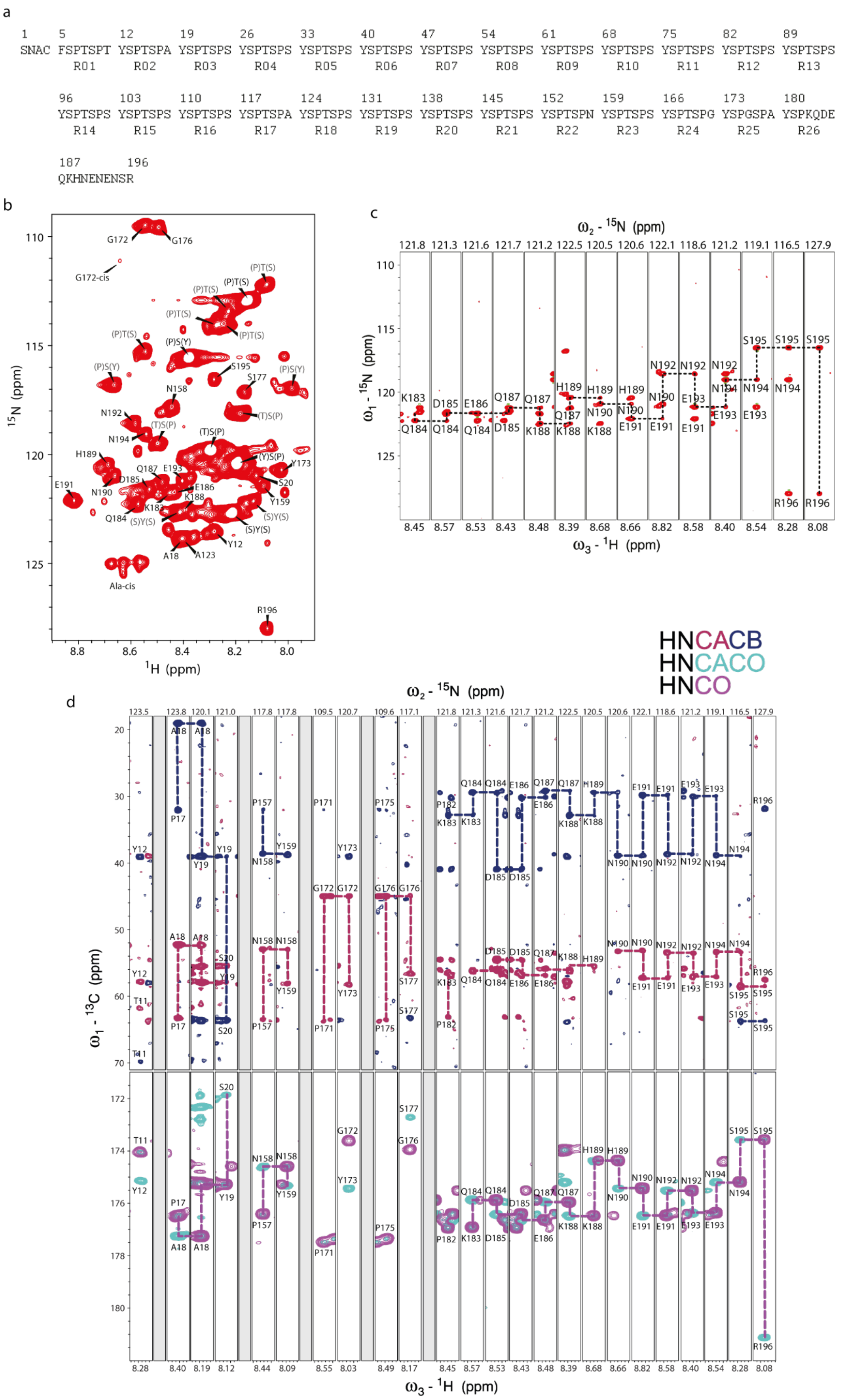

Supplementary Fig. 5 │ NMR resonance assignment of yCTD. a, Amino acid sequence of the yCTD protein used in the current study. **b,** ^{1}H-^{15}N-HSQC spectrum of yCTD with residue-specific assignments. The spectrum was recorded at 5 °C. Groups of overlapped peaks are marked in grey using sequential motifs with preceding and succeeding residues in brackets and assigned residue types in between. Groups of minor peaks are thought to be resonances appearing from *cis/trans*-isomerization of proline residues. **c,** Strip-assisted assignment of the triple-resonance experiment (H)N(CA)NNH. **d,** Resonance strips from 3D HNCACB (upper part), HN(CA)CO, and HNCO (lower part) of yCTD. Residue-specific assignments are indicated.

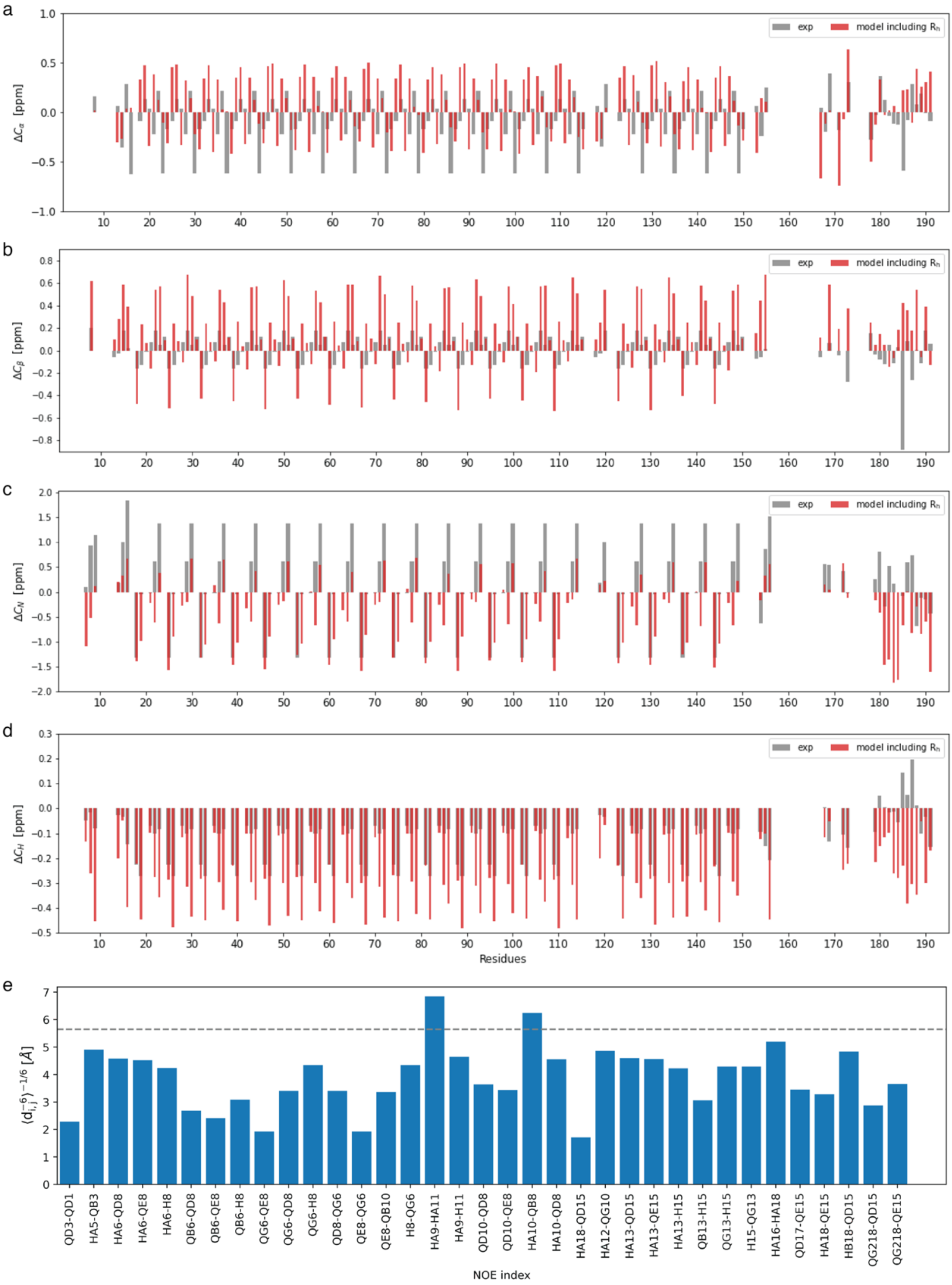

Supplementary Fig. 6 │ Comparison of experimental NMR parameters with values back-calculated from HCG models of yCTD. a-d, NMR chemical shifts in yCTD. **e,** Distance restraints derived from experimental NOE cross peaks of 3R-CTD with average distances observed in a 21-residue segment composed of three YSPTSPS repeats extracted from the HCG ensemble of yCTD. Except for two distance restraints, all experimental NOE restraints are fulfilled according to a cut-off of 5.65 Å (dashed line).

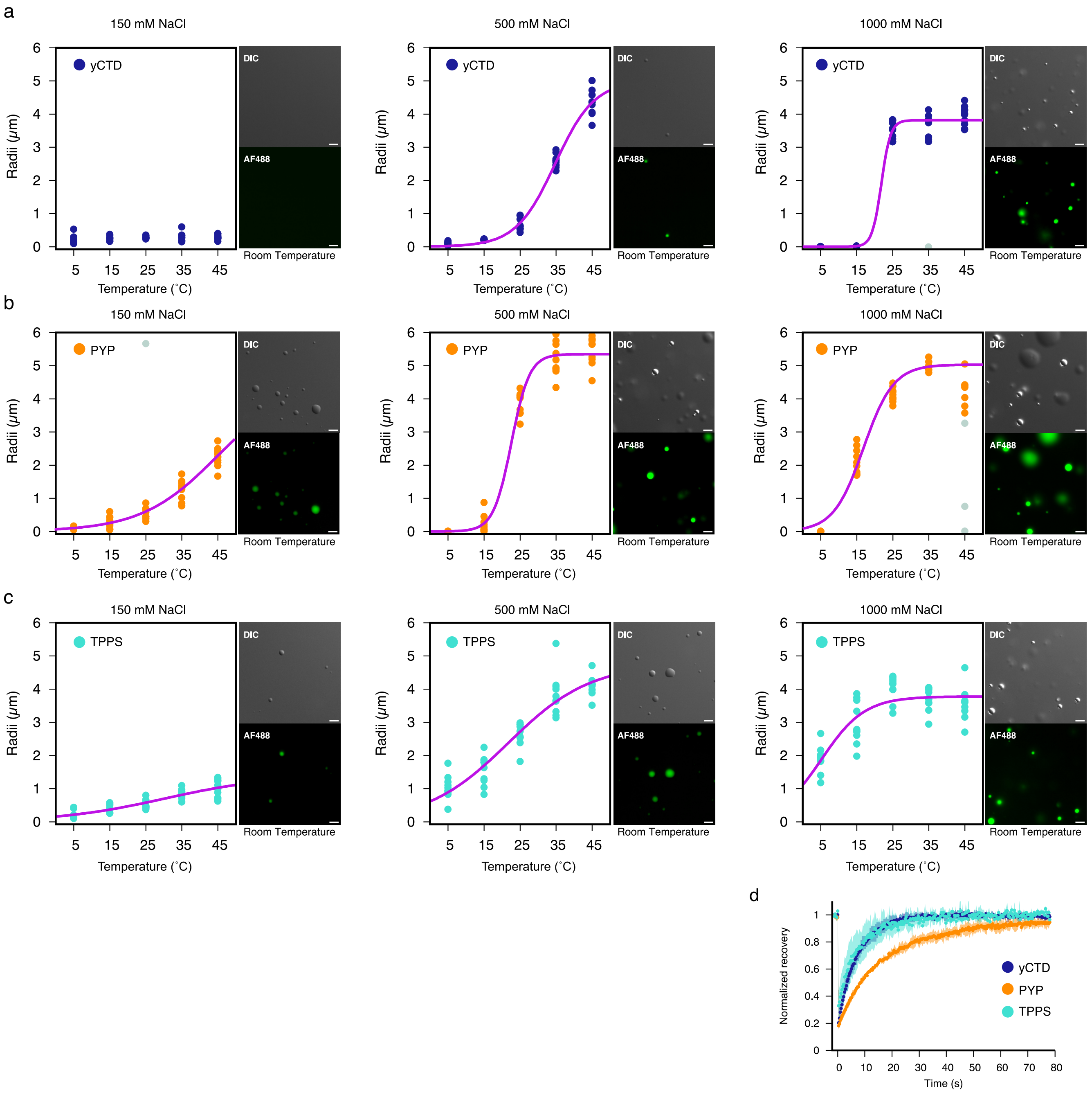

Supplementary Fig. 7 | Phase separation of designed yCTD variants. a-c, DLS plots, and micrographs for the formation of liquid-like droplets of yCTD (a), PYP (b), and TPPS (c). Each protein was tested at 100 µM, MES 25 mM, TCEP 1 mM, and dextran 5% w/v at pH 6.2. Purple curves indicate the sigmoidal fit to the experimental data. The salt concentration is indicated at the top of each DLS plot in the panels. Micrographs were acquired at room temperature (~22˚C) and are representative of 3 independent biological replicates. Scale bars, 5 µm. **d,** FRAP experiments (n=3) illustrating the diffusivity of the variants in the high salt content condition (1M).

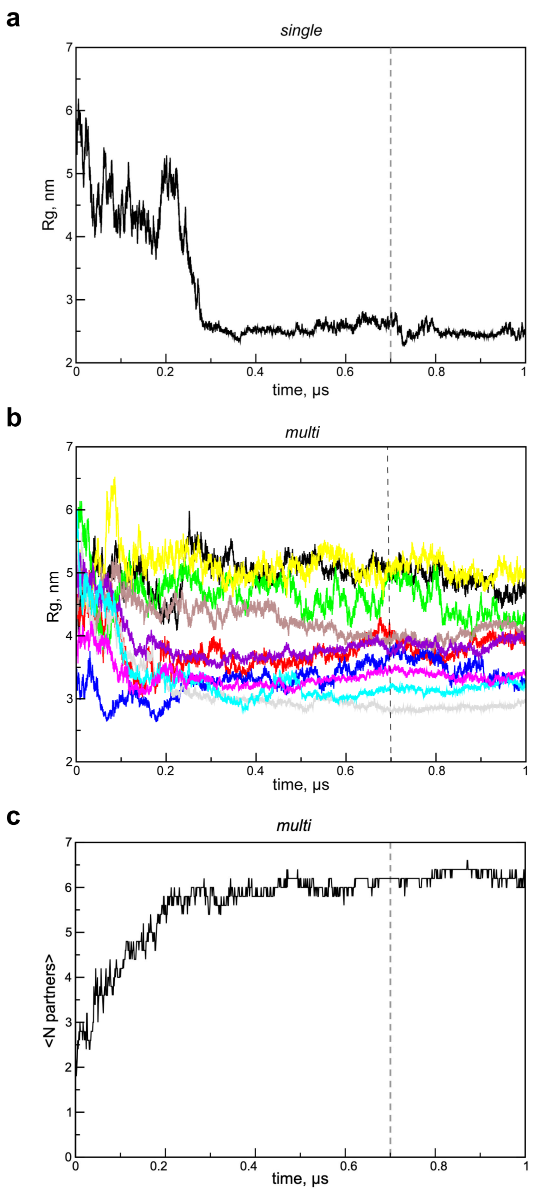

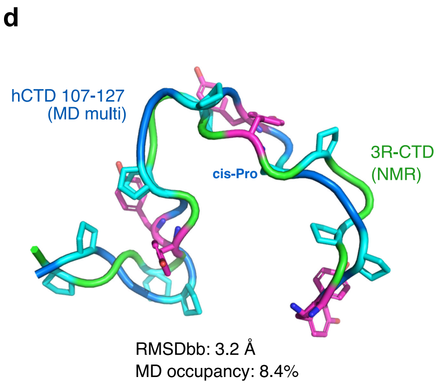

e

multi

	frac	enr
P-S	0,14	0,74
P-Y	0,14	1,8
S-Y	0,12	1,3
P-T	0,08	0,9
P-P	0,08	1,04
S-T	0,08	0,72
T-Y	0,07	1,63
S-S	0,06	0,55
Y-Y	0,05	2,61
T-T	0,02	0,9
rest	0,164	

f

single

	frac	enr
P-S	0,17	0,93
S-Y	0,11	1,23
S-S	0,11	0,96
P-Y	0,09	1,25
S-T	0,09	0,85
P-T	0,09	1,02
P-P	0,08	1,02
T-Y	0,04	0,98
Y-Y	0,04	1,98
T-T	0,04	1,38
rest	0,135	

Supplementary Fig. 8 | Analysis of conformational preferences and contact statistics in all-atom MD simulations of hCTD. **a**, Radius of gyration (Rg) of hCTD as a function of time in single-copy simulation. **b**, Rg values of individual hCTD conformers as a function of time in multi-copy simulation. **c**, the average number of interacting partners per conformer (<N $_{\text{partners}}$>) as a function of time in multi-copy simulation. The dotted lines in **a-c** capture the time point after which analysis of contact statistics was performed. **d**, Comparison of the closest conformation of a 21-mer fragment in the full-length hCTD in multi-copy MD simulations (blue) to one of the Rosetta ensemble conformations of 3R-CTD (green). Proline and tyrosine residues are shown in cyan and magenta, respectively. Backbone RMSD between the two structures and the occupancy of the corresponding conformational cluster (cutoff of 1 Å in backbone RMSD) are indicated. **e-f**, Absolute fractions (frac) and enrichments (enr) over the sequence composition-dependent background for the top 10 pairwise contacts in multi-copy (e, intermolecular) and single-copy (f, intramolecular) MD simulations.

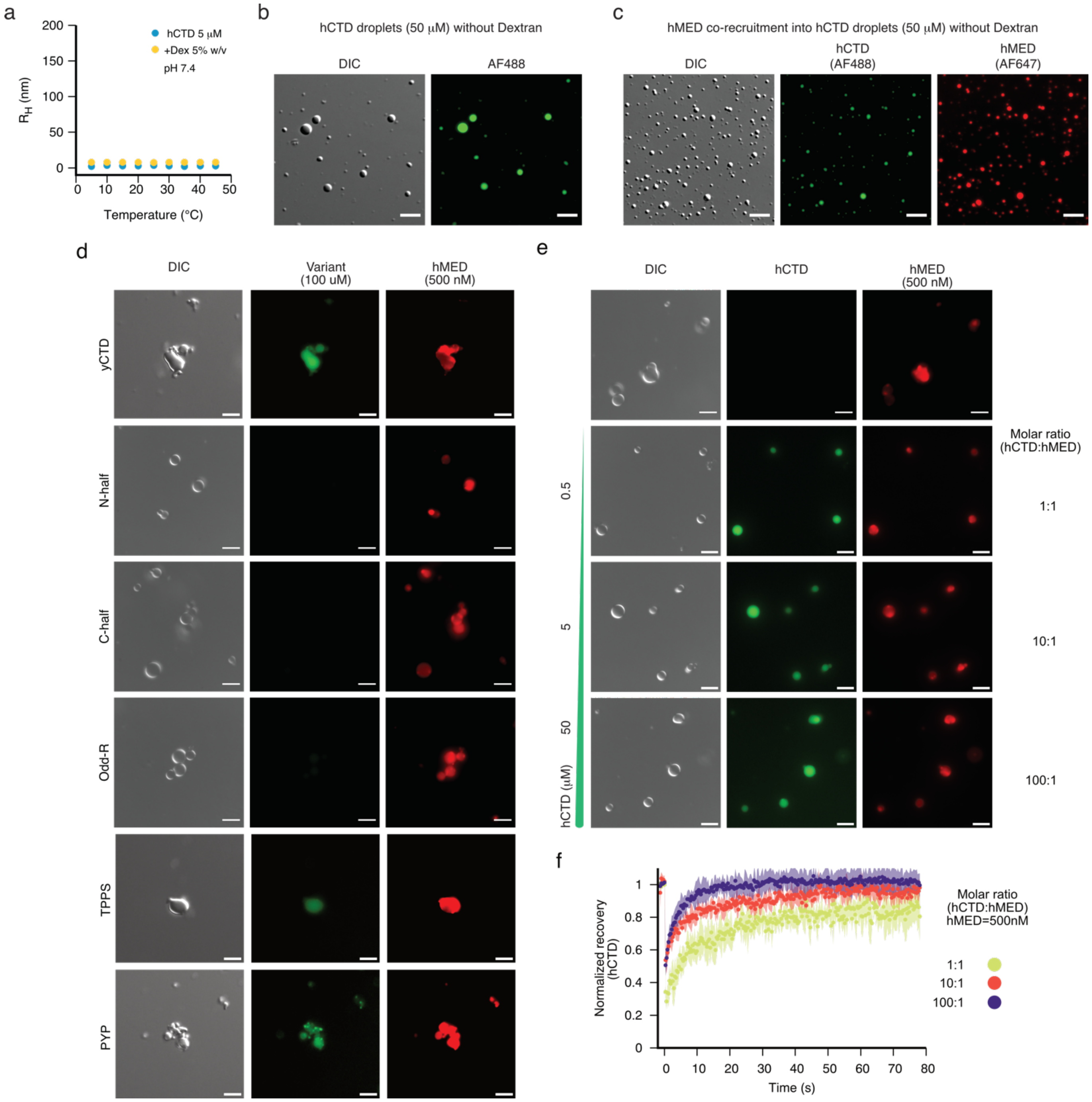

Supplementary Fig. 9 |Phase separation of hCTD and co-recruitment with hMED. a, Hydrodynamic radii R_h of hCTD (5 µM hCTD, HEPES 25 mM, NaCl 150 mM, TCEP 1 mM and pH 7.4) by DLS at increasing temperatures. **b,** Micrographs for self-coacervation of 50 µM hCTD in the same conditions (without dextran) as panel (a). hCTD was fluorescently labeled with Alexa Flour 488 (AF488). **c,** hMED recruitment into hCTD droplets (HEPES 25 mM, NaCl 150 mM, TCEP 1 mM and pH 7.4). hMED was fluorescently labeled with Alexa Flour 647 (AF647). **d,** Micrographs investigating co-recruitment of yCTD variants (100 µM) and the human Mediator complex (500 nM) during phase separation (5 % w/v dextran, 25 mM HEPES, 150 mM NaCl, 1mM TCEP at pH 7.4). CTD variants were fluorescently labeled with Alexa 488 (green) and hMED was labeled with Alexa 647 (red). **e,** Co-recruitment of hCTD and hMED during phase separation at different molar ratios. Conditions are identical to those in panel (a). **f,** FRAP (mean ± std) of hCTD at different molar ratios of hCTD and hMED (500 nM). Micrographs are representative of 3 independent biological replicates. Scale bar, 5 µm.

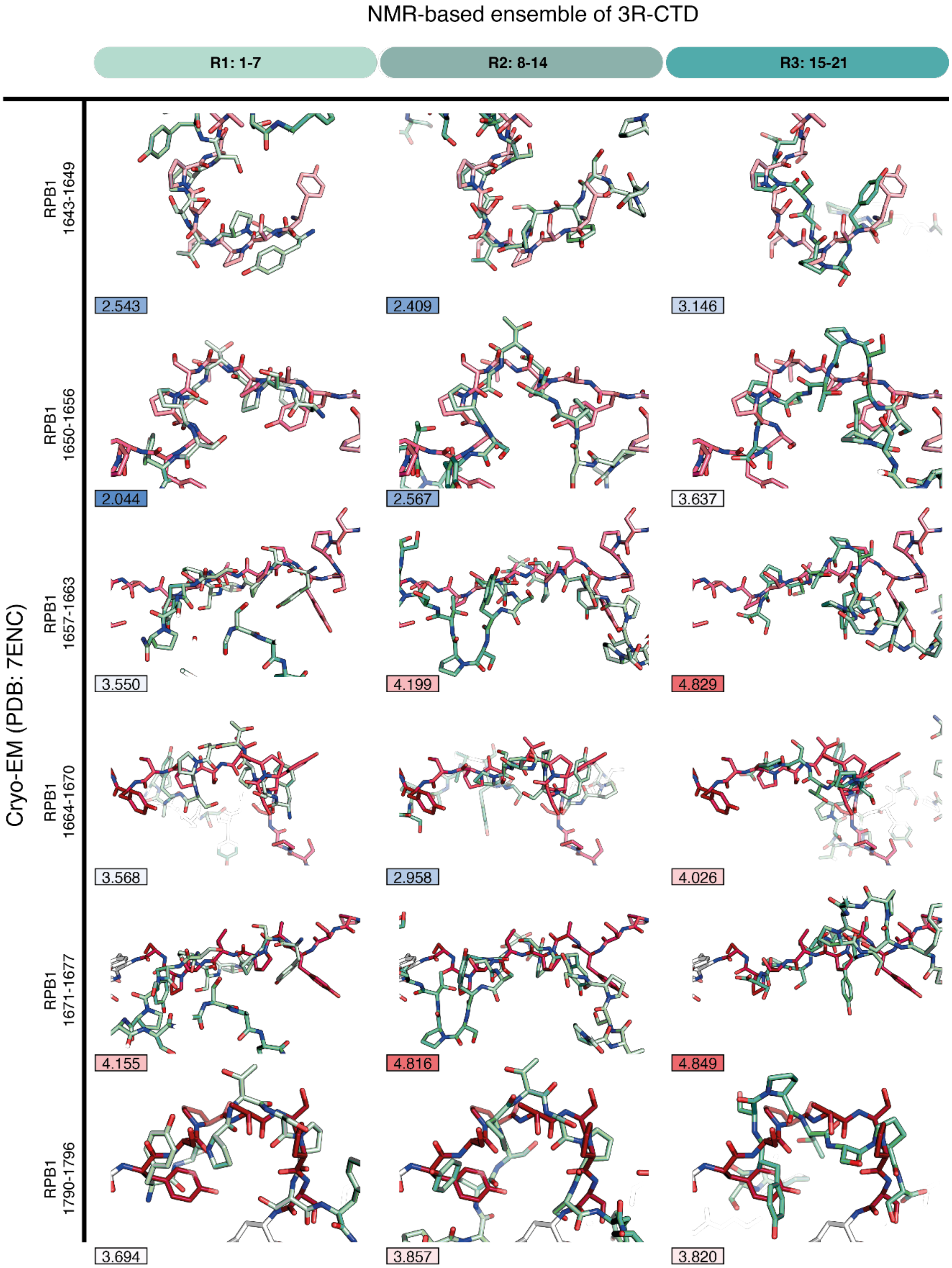

Supplementary Fig. 10 | Comparison of CTD conformations bound to the Mediator complex with conformations of canonical YSPTSPS repeats observed in the ensemble of NMR-based structures of 3R-CTD. Columns correspond to the NMR-based models (ensemble), while rows represent heptad repeats resolved by cryo-EM (PDB ID: 7ENC). Numbers enclosed in boxes represent the rmsd (increasing rmsd from blue to red).

Supplementary Table 1 | Details for the molecular dymanic simulations of hCTD.

condition	simulation box size	atoms per box	hCTD molecules per box	water molecules per box	NaCl concentration	simulation length
**single-copy**	20.0 x 20.0 x 20.0 nm^3	1052851	1	261515	0.15 M	1 ms
**multi-copy**	19.9 x 19.9 x 19.9 nm^3	1034428	10	244883	0.15 M	1 ms
