# Supplementary material for: Driving forces behind phase separation of the carboxy-terminal domain of RNA polymerase II
Source: Nat Commun. 2023 Sep 25;14:5979. doi: 10.1038/s41467-023-41633-8 (PMC10519987; doi:10.1038/s41467-023-41633-8)
Supplement: Supplementary file 3 — Description of Additional Supplementary Files [file 41467_2023_41633_MOESM3_ESM.pdf]

Description of Additional Supplementary Files

File name: Supplementary Data 1

Description: GROMACS simulation input files as well as the coordinates of the first and last simulation snapshots.
