# Supplementary material for: Driving forces behind phase separation of the carboxy-terminal domain of RNA polymerase II
Source: Nat Commun. 2023 Sep 25;14:5979. doi: 10.1038/s41467-023-41633-8 (PMC10519987; doi:10.1038/s41467-023-41633-8)
Supplement: Supplementary file 5 — Reporting Summary [file 41467_2023_41633_MOESM5_ESM.pdf]

nature research

Corresponding author(s): Markus Zweckstetter

Last updated by author(s): Aug 31, 2023

Reporting Summary

Nature Research wishes to improve the reproducibility of the work that we publish. This form provides structure for consistency and transparency in reporting. For further information on Nature Research policies, see our Editorial Policies and the Editorial Policy Checklist.

Statistics

For all statistical analyses, confirm that the following items are present in the figure legend, table legend, main text, or Methods section.

n/a	Confirmed	
☐	☒	The exact sample size (*n*) for each experimental group/condition, given as a discrete number and unit of measurement
☐	☒	A statement on whether measurements were taken from distinct samples or whether the same sample was measured repeatedly
☒	☐	The statistical test(s) used AND whether they are one- or two-sided<br>*Only common tests should be described solely by name; describe more complex techniques in the Methods section.*
☒	☐	A description of all covariates tested
☒	☐	A description of any assumptions or corrections, such as tests of normality and adjustment for multiple comparisons
☐	☒	A full description of the statistical parameters including central tendency (e.g. means) or other basic estimates (e.g. regression coefficient) AND variation (e.g. standard deviation) or associated estimates of uncertainty (e.g. confidence intervals)
☒	☐	For null hypothesis testing, the test statistic (e.g. *F*, *t*, *r*) with confidence intervals, effect sizes, degrees of freedom and *P* value noted<br>*Give P values as exact values whenever suitable.*
☒	☐	For Bayesian analysis, information on the choice of priors and Markov chain Monte Carlo settings
☒	☐	For hierarchical and complex designs, identification of the appropriate level for tests and full reporting of outcomes
☒	☐	Estimates of effect sizes (e.g. Cohen's *d*, Pearson's *r*), indicating how they were calculated

Our web collection on statistics for biologists contains articles on many of the points above.

Software and code

Policy information about availability of computer code

Data collection	NMR data were acquired using Topspin versions 3.5pl7 or 4.0.8. DLS data were acquired with DYNAMICS v7.10.0.23. Optical microscope pictures are acquired with Leica Application Suite X.
Data analysis	NMR data were processed with NMRPipe 10.1 Rev 2019.217.11.55 64-bit for Mac and analyzed using Sparky 1.414. Secondary structure and phi/psi angles were calculated with TALOS-N. MacPyMOL v1.7.6.0 was used to build models and representations. Optical microscope micrographs and FRAP experiments were analyzed with ImageJ v1.52q. DLS data were analyzed with DYNAMICS v7.10.0.23. Rosetta models were validated with MolProbity 4.5. Gnuplot Version 5.4pl3 was used for plotting data.

For manuscripts utilizing custom algorithms or software that are central to the research but not yet described in published literature, software must be made available to editors and reviewers. We strongly encourage code deposition in a community repository (e.g. GitHub). See the Nature Research guidelines for submitting code & software for further information.

Data

Policy information about availability of data

All manuscripts must include a data availability statement. This statement should provide the following information, where applicable:

- Accession codes, unique identifiers, or web links for publicly available datasets
- A list of figures that have associated raw data
- A description of any restrictions on data availability

PDB code(s) cited (7ENC) are publicly available in the Protein Data Bank. All data that support the findings of this study are available from the corresponding authors upon reasonable request.

Field-specific reporting

Please select the one below that is the best fit for your research. If you are not sure, read the appropriate sections before making your selection.

☒ Life sciences ☐ Behavioural & social sciences ☐ Ecological, evolutionary & environmental sciences

For a reference copy of the document with all sections, see nature.com/documents/nr-reporting-summary-flat.pdf

Life sciences study design

All studies must disclose on these points even when the disclosure is negative.

Sample size	DLS data were acquired with n≥3 per batch to observe natural tendencies. Multiple micrographs were collected (n≥5 per condition) and selected based on the central tendency of number of bodies in the pictures.
Data exclusions	No data have been excluded while reporting this study.
Replication	NMR data was acquired with multiple batches of hCTD (natural abundance = na & U-[15N]), yCTD (na, U-[15N] and U-[15N, 13C] labeled) and Y1S variants (na) observing reproducible results. DLS data showed same results among batches. Micrographs from different batches were processed with an unified adjust of contrast and brightness showing a reliable comparison.
Randomization	None for protein preparation. Protein batches were prepared following the protocols described in the methods section. Micrographs have been taken in multiple sites to monitor the average tendency of the systems for phase separation.
Blinding	Phase separation has been tested in highly pure proteins by two orthogonal techniques in order to reduce the risk of bias. NMR is interpreted with the common chemical shifts from the Biological Magnetic Resonance Bank.

Reporting for specific materials, systems and methods

We require information from authors about some types of materials, experimental systems and methods used in many studies. Here, indicate whether each material, system or method listed is relevant to your study. If you are not sure if a list item applies to your research, read the appropriate section before selecting a response.

Materials & experimental systems

n/a	Involved in the study
☒	☐ Antibodies
☒	☐ Eukaryotic cell lines
☒	☐ Palaeontology and archaeology
☒	☐ Animals and other organisms
☒	☐ Human research participants
☒	☐ Clinical data
☒	☐ Dual use research of concern

Methods

n/a	Involved in the study
☒	☐ ChIP-seq
☒	☐ Flow cytometry
☒	☐ MRI-based neuroimaging
